# Non-invasive Neurally Adjusted Ventilatory Assist (NIV-Nava): Four-Year Experience in a Level III Neonatal Intensive Care Unit

**DOI:** 10.7759/cureus.116942

**Published:** 2026-09-25

**Authors:** Madalena Ferreira, Margarida Roquette, Inês Belo, Cátia R Correia, Anabela Salazar

**Affiliations:** 1 Neonatology, São Francisco Xavier Hospital, Western Lisbon Local Health Unit, Lisbon, PRT; 2 Pediatrics, Cascais Hospital Dr. José de Almeida, Lisbon, PRT

**Keywords:** neonatal respiratory support, neonatology, niv-nava, non-invasive ventilation, prematurity

## Abstract

Introduction

Non-invasive neurally adjusted ventilatory assist (NIV-NAVA) is a ventilation mode that uses the electrical activity of the diaphragm to synchronize respiratory support with the patient’s spontaneous effort. It has emerged as a promising non-invasive modality for neonates. We aimed to evaluate the clinical success of NIV-NAVA in a neonatal intensive care unit (NICU) setting. To our knowledge, this is the first study in Portugal to describe the experience with NIV-NAVA in a level III NICU.

Methods

We conducted an observational retrospective cohort study through a medical record review over four years (January 2021 to December 2024). All newborns admitted to our NICU who were ventilated using NIV-NAVA were included. Descriptive statistical analysis was performed on demographic and diagnostic data. Ventilatory and gasometrical parameters, including fraction of inspired oxygen (FiO₂), pH, and partial pressure of carbon dioxide (pCO₂), were evaluated before and after NIV-NAVA initiation. A respiratory severity score (RSS) was calculated at baseline to assess the severity of respiratory disease. The primary outcome was the proportion of NIV-NAVA episodes not requiring subsequent endotracheal intubation, and success was defined as the avoidance of subsequent intubation.

Results

A total of 36 NIV-NAVA episodes were recorded across 28 newborns, with a mean gestational age of 26 weeks and a mean birth weight of 822g. All newborns were diagnosed with prematurity, respiratory distress syndrome, and bronchopulmonary dysplasia. The mean baseline RSS was 3.7, reflecting the respiratory disease burden at NIV-NAVA initiation. NIV-NAVA was initiated following extubation in 83.3% of cases and as therapeutic escalation in 16.7%. Success was achieved in 58.3% of all episodes (60% in the post-extubation group and 50% in the escalation group). No statistically significant differences were observed in mean FiO₂ or pCO₂ values before and after NIV-NAVA.

Discussion

NIV-NAVA success was observed in the majority of newborns, including extremely low birth weight infants with clinically relevant respiratory disease burden. While NIV-NAVA offers significant theoretical advantages regarding patient-ventilator synchronization, published evidence in the NICU remains limited.

Conclusion

Our findings support the use of NIV-NAVA as a feasible non-invasive ventilatory modality in preterm infants, particularly for post-extubation respiratory support, although further randomized controlled trials are warranted to better establish its role and efficacy within the neonatal population.

## Introduction

Non-invasive ventilation (NIV) has become a cornerstone of neonatal respiratory distress management, significantly improving patient outcomes by reducing complications associated with invasive mechanical ventilation [1]. Current guidelines for primary and secondary respiratory support in preterm infants have shifted from routine endotracheal intubation to early NIV. This transition is driven by improved safety and superior clinical outcomes, including a reduced risk of bronchopulmonary dysplasia (BPD) and mortality [2-4].

While traditional modalities such as nasal continuous positive airway pressure (nCPAP) and nasal intermittent positive pressure ventilation (NIPPV) are well-established, non-invasive neurally adjusted ventilatory assist (NIV-NAVA) has been emerging as a new approach. This modality is particularly promising for preterm infants and those with transient tachypnea of the newborn (TTN) [4-8]. NIV-NAVA works by using a modified nasogastric tube with electrodes that detect the electrical activity of the diaphragm (Edi). These signals allow the ventilator to deliver support in proportion to the patient’s respiratory effort, ensuring superior synchronization. By responding to the infant’s neural respiratory drive in real-time, NIV-NAVA adjusts peak inspiratory pressure (PIP), mean airway pressure, tidal volume, and inspiratory times (Ti), fostering a more physiological breathing pattern [5-7, 9].

Preterm infants are particularly vulnerable to respiratory failure due to lung immaturity, surfactant deficiency, and erratic breathing patterns resulting from underdeveloped neural breathing control. In this context, NIV-NAVA may offer distinct advantages over conventional NIV methods, which rely on pre-set pressures or flow rates and may be less effective in ensuring optimal synchronization, potentially leading to barotrauma, air leaks, and volutrauma [9-10].

Recent evidence suggests that NIV-NAVA is a safe and feasible alternative, offering short-term physiological benefits, including lower PIP, reduced fraction of inspired oxygen (FiO₂) requirements, fewer and shorter desaturation episodes, and decreased work of breathing, with no significant adverse events reported to date [5, 7, 11-12]. Furthermore, recent randomized controlled trials and meta-analyses indicate that NIV-NAVA may reduce extubation failure rates compared to nCPAP or NIPPV. However, the certainty of evidence remains limited due to small sample sizes and heterogeneity in study designs, and further large-scale trials are still lacking [8, 13-14].

While NIV-NAVA theoretically optimizes synchronization and appears to be a safe and effective alternative to conventional NIV modalities, particularly for post-extubation support, robust data on long-term outcomes are still needed [7-8, 13-14].

This study sought to evaluate the clinical experience and success of NIV-NAVA in a level III neonatal intensive care unit (NICU). We hypothesized that NIV-NAVA would provide effective non-invasive respiratory support, allowing avoidance of subsequent endotracheal intubation in a substantial proportion of episodes. We further explored outcomes according to the indication for NIV-NAVA initiation: post-extubation support or escalation from other non-invasive ventilation modalities.

## Materials and methods

Study design and population

We conducted a retrospective observational cohort study through a medical record review over four years (January 1st, 2021, to December 31st, 2024). The study population included all neonates admitted to our level III NICU who were ventilated with NIV-NAVA during their hospitalization. Patients transferred and those with incomplete medical records were excluded.

Data collection

Maternal and neonatal demographic and clinical data, as well as ventilatory and gasometrical parameters, were collected from electronic medical records. Maternal data included antenatal corticosteroid administration for fetal maturation (classified as complete, incomplete, or absent). Neonatal data encompassed birth-related parameters, surfactant administration, and therapies such as caffeine citrate and postnatal corticosteroids. Major neonatal morbidities were recorded, including prematurity-related complications, respiratory distress syndrome (RDS), BPD, sepsis, pneumonia, and peri-intraventricular hemorrhage (PIVH).

Ventilatory and gasometric parameters

Ventilatory modes and parameters, including FiO2, positive end-expiratory pressure (PEEP), and gasometric data [pH, partial pressure of carbon dioxide (pCO_2_)], were assessed immediately before and after NIV-NAVA initiation. We collected demographic data at NIV-NAVA initiation (chronological age, postmenstrual age, and weight), initial and maximum NIV-NAVA parameters (NIV-NAVA level, PEEP), and total duration of NIV-NAVA support and hospital stay.

Definitions and outcomes

Infants requiring NIV-NAVA on more than one occasion during the same admission were analyzed as distinct episodes. The primary outcome was the proportion of NIV-NAVA episodes not requiring subsequent endotracheal intubation. NIV-NAVA success was defined as avoidance of endotracheal intubation, whereas NIV-NAVA failure was defined as the requirement for endotracheal intubation following NIV-NAVA initiation. In cases of NIV-NAVA failure, we investigated the presence of active infection (sepsis or pneumonia) from 48 hours before to 72 hours after NIV-NAVA initiation. Sepsis was defined by clinical signs in conjunction with microbiological confirmation (positive blood cultures). Pneumonia was defined by clinical respiratory distress associated with radiographic evidence of pulmonary infiltrates. Given the retrospective design, all decisions regarding ventilatory management and intubation were made by the attending neonatologist based on clinical judgment. To standardize the assessment of respiratory disease severity at NIV-NAVA initiation, the respiratory severity score (RSS) was calculated [15].

- For invasive ventilation: $\mathrm{RSS} = \left(\frac{\mathrm{PIP}}{3} + \frac{2\,\mathrm{PEEP}}{3}\right) \times \mathrm{FiO}_{2}$

- For non-invasive ventilation: $\mathrm{RSS} = \frac{\mathrm{MAP}}{\mathrm{PEEP}} \times \mathrm{FiO}_{2}$

FiO₂ was expressed as a decimal fraction ranging from 0.21 to 1.00.

Statistical analysis

Statistical analysis was performed using SPSS version 22 (IBM Corp., Armonk, USA). As this was a retrospective single-center study including all eligible NIV-NAVA episodes during the predefined four-year period, no formal a priori sample size calculation was performed. Descriptive statistical analysis was performed for all collected data, and continuous variables were summarized using mean and standard deviation (SD) or median and range, as appropriate. Differences between pre- and post-NIV-NAVA FiO₂, PEEP, pH, and pCO₂ values were analyzed using paired Student’s t-tests. Statistical significance was considered to be a p-value <0.05.

## Results

Patient characteristics

During the study period, a total of 1,074 infants were admitted to the NICU, of whom 28 underwent ventilation with NIV-NAVA, accounting for 36 registered episodes. The studied cohort had a mean gestational age of 26 weeks and a mean birth weight of 822 grams, with a median hospital stay of 90 days. All infants were premature and were diagnosed with RDS and BPD. BPD was defined as the requirement for supplemental oxygen and/or respiratory support for at least 28 days of life. PIVH was found in 75% (n=21) of infants, and 67.9% (n=19) had sepsis confirmed by positive blood culture (Table 1).

**Table 1:** Participants demographic data. BPD: bronchopulmonary dysplasia; EOS: early-onset sepsis; LOS: late-onset sepsis; n: number of infants; NIV-NAVA: non-invasive neurally adjusted ventilatory assist; PIVH: peri-intraventricular haemorrhage; RDS: respiratory distress syndrome. *Two infants presented with both EOS and LOS.

Demographic data	Total - Number of infants (n=28)
Male infants; Female infants - n (%)	10 (35.7%); 18 (64.3%)
		Gestational age in weeks and days - median (range)	26 weeks + 4 days (24 weeks + 2 days - 31 weeks +3 days)
Birth weight in grams - median (range)	804g (530-1220g)
		Very low birth weight (1000–1499 g) - n (%)	5 (17.9%)
		Extremely low birth weight (<1000 g) - n (%)	23 (82.1%)
Vaginal delivery - n (%)	4 (14.3%)
Caesarean section - n (%)	24 (85.7%)
Apgar score at 1 min; 5 min; 10 min - median (range)	7 (3-9); 8 (6-9); 9 (8-9)
Antenatal corticotherapy for fetal maturation - Complete; Incomplete - n (%)	19 (67.9%); 9 (32.1%)
Surfactant - n (%)	28 (100%)
Caffeine use - n (%)	28 (100%)
Postnatal corticotherapy - n (%)	14 (50%)
Prematurity - n (%)	28 (100%)
Respiratory distress syndrome (RDS) - n (%)	28 (100%)
Bronchopulmonary dysplasia (BPD) - n (%)	28 (100%)
Sepsis - n (%)	19/28 (67.9%)
Early-onset sepsis (EOS)* - n (%)	2/28 (7.1%)
Late-onset sepsis (LOS)* - n (%)	19/28 (67.9%)
Pneumonia - n (%)	8 (28.6%)
Peri-intraventricular haemorrhage (PIVH) - n (%)	21 (75%)
PIVH grade 1; grade 2; grade 3; grade 4 - n (%)	10 (35.7%); 6 (21.4%); 5 (17.9%); 0
Survival rate - n (%)	26 (92.9%)

Clinical management and NIV-NAVA initiation

Antenatal corticosteroids for fetal maturation were administered in all cases (68% complete; 32% incomplete). Furthermore, all infants received surfactant and caffeine citrate. Postnatal dexamethasone, following the low-dose dexamethasone: a randomized trial (DART) protocol, was administered to 50% of the cohort.

NIV-NAVA was started following extubation in 83.3% of the episodes (n=30/36); of these, half had transitioned from conventional mechanical ventilation and the other half from invasive neurally adjusted ventilatory assist (NAVA). The remaining 16.7% of episodes (n=6/36) involved therapeutic escalation from CPAP. At the time of NIV-NAVA initiation, infants had a mean chronological age of 41.5 days, a mean post-menstrual age (PMA) of 31 weeks, and an average weight of 1584.8 grams. The median duration of NIV-NAVA support was nine days. The mean RSS before NIV-NAVA initiation was 3.7, reflecting the respiratory disease burden.

NIV-NAVA success and failure

NIV-NAVA ventilation was successful in the majority of episodes (58.3%, n= 21/36). Within this successful group, eight episodes were transitioned to CPAP and 13 to high-flow nasal cannula (HFNC) following NIV-NAVA. NIV-NAVA failed in the remaining 41.7% of episodes (n=15/36), requiring subsequent intubation; of these, 11 episodes were transitioned to conventional mechanical ventilation and four to invasive NAVA. When analyzed by clinical indication, the success rate was 60% (n=18/30) in the post-extubation group and 50% (n = 3/6) in the escalation group.

Analysis of NIV-NAVA failure and infection

Among the 15 cases of NIV-NAVA failure requiring intubation, evidence of active infection from 48 hours before to 72 hours after NIV-NAVA was identified in 46.7% (n=7/15) of episodes. Specifically, four neonates developed sepsis (26.7%, n=4/15), while three were diagnosed with pneumonia (20%, n=3/15) at the time of NIV-NAVA initiation. Detailed clinical and ventilatory data before and after NIV-NAVA are presented in Table 2.

**Table 2:** Baseline clinical data, ventilatory parameters and clinical outcomes of NIV-NAVA episodes. FiO2: fraction of inspired oxygen; n: number of episodes; N/A: not applicable; NAVA: neurally adjusted ventilatory assist; nCPAP: nasal continuous airway pressure; NIV-NAVA: non-invasive neurally adjusted ventilatory assist; PEEP: positive end-expiratory pressure; PIP: peak inspiratory pressure.

	Post-extubation group (n=30)	Escalation group (n=6)	Total number of episodes (n=36)
Chronological age at NIV-NAVA initiation, days - median (range)	41.5 (5-190)	37 (10-94)	41.5 (5-190)
Postmenstrual age at NIV-NAVA initiation, weeks+days - median (range)	31+4 (27+3-43+0)	30+1 (28+6-32+3)	31+3 (27+3-43+0)
Weight at NIV-NAVA initiation, grams - median (range)	1206 (772-4401)	1115 (885-2283)	1206 (772-4401)
Pre-NIV-NAVA respiratory support: nCPAP - n (%)	N/A	6 (100%)	6 (17%)
Pre-NIV-NAVA respiratory support: NAVA - n (%)	15 (50%)	N/A	15 (42%)
Pre-NIV-NAVA respiratory support: conventional mechanical ventilation - n (%)	15 (50%)	N/A	15 (42%)
Baseline respiratory severity score - median (range)	2.7 (1-13.1)	2.8 (1.7-6.4)	2.7 (1-13.1)
Pre-NIV-NAVA FiO_2_, % - median (range)	32.5 (21-75)	35 (21-80)	32.5 (21-80)
Pre-NIV-NAVA PIP, cmH₂O - median (range)	20 (10-40)	N/A	20 (10-40)
Pre-NIV-NAVA PEEP, cmH₂O - median (range)	6 (5-8)	6.5 (5.5-7.5)	6.3 (5-8)
Initial NIV-NAVA level, cmH₂O/µV - median (range)	2.0 (1.5–4.5)	2 (1.8–4.5)	2 (1.5-4.5)
Initial NIV-NAVA FiO_2_, % - median (range)	45 (21-85)	30 (28–80)	40 (21-85)
Initial NIV-NAVA PEEP, cmH₂O - median (range)	6.5 (5-8)	6.5 (5.5-8)	6.5 (5-8)
Maximum NIV-NAVA level, cmH₂O/µV - median (range)	2.5 (1.5–6.5)	2.0 (1.8–4.5)	2.5 (1.5–6.5)
Maximum NIV-NAVA FiO_2_, % - median (range)	47.5 (21-100)	50 (28-85)	47.5 (21-100)
Maximum NIV-NAVA PEEP, cmH₂O - median (range)	7 (5.5-10)	7 (5.5-8)	7 (5.5-10)
NIV-NAVA duration, days - median (range)	10 (2-18)	9 (1-29)	9 (1-29)
Post-NIV-NAVA respiratory support: nCPAP - n (%)	7 (23.3%)	1 (16.7%)	8 (22%)
Post-NIV-NAVA respiratory support: HFNC - n (%)	11 (36.7%)	2 (33.3%)	13 (36%)
Post-NIV-NAVA respiratory support: NAVA - n (%)	3 (10%)	1 (16.7%)	4 (11%)
Post-NIV-NAVA respiratory support: Conventional mechanical ventilation - n (%)	9 (30%)	2 (33.3%)	11 (31%)
NIV-NAVA success (no subsequent endotracheal intubation) - n (%)	18 (60%)	3 (50%)	21 (58.3%)
NIV-NAVA failure (subsequent endotracheal intubation) - n (%)	12 (40%)	3 (50%)	15 (41.7%)
Active infection (within the predefined peri-NIV-NAVA period) - n (%)	5/12 (41.7%)	2/3 (66.7%)	7/15 (46.7%)
Sepsis (within the predefined peri-NIV-NAVA period) - n (%)	3/12 (25%)	1/3 (33.3%)	4/15 (26.7%)
Pneumonia (within the predefined peri-NIV-NAVA period) - n (%)	2/12 (16.7%)	1/3 (33.3%)	3/15 (20%)
Total length of hospital stay, days - median (range)	93.5 (7-283)	90 (7-283)	90 (7-283)

Physiological and gasimetric parameters

The evaluation of ventilatory parameters before and after NIV-NAVA (Table 3) revealed no statistically significant changes. The mean FiO2 remained stable (40.9% vs 42%, respectively, p-value 0.58), as did the mean pCO_2_ levels (55.5 mmHg vs. 57.2 mmHg, respectively, p-value 0.30).

**Table 3:** Ventilatory and gasometric parameters before and after initiating NIV-NAVA. FiO2: fraction of inspired oxygen; NIV-NAVA: non-invasive neurally adjusted ventilatory assist; pCO_2_: partial pressure of carbon dioxide; PEEP: positive end-expiratory pressure; SD: standard deviation. * A p-value <0.05 was considered statistically significant.

	Before NIV-NAVA (mean ± SD)	After NIV-NAVA (mean ± SD)	Mean difference	p-value*
FiO₂ (%)	40.9 ± 17.3	42.0 ± 17.1	+1.17	0.58
PEEP (cmH₂O)	6.44 ± 0.85	6.51 ± 1.29	+0.13	0.54
pH	7.381 ± 0.060	7.379 ± 0.055	–0.0014	0.91
pCO₂ (mmHg)	55.5 ± 9.3	57.2 ± 12.6	+1.70	0.30

## Discussion

To our knowledge, this represents the first cohort study in Portugal to evaluate NIV-NAVA experience in a level III NICU over a four-year period. Our primary objective was to assess the success of NIV-NAVA, defined as the avoidance of subsequent endotracheal intubation. We found that the majority of episodes (58.3%; n=21/36) were successfully managed without the need for subsequent invasive ventilation. When stratified by clinical indication, the success rate was higher in the post-extubation group (60%; n=18/30) compared to the escalation group (50%; n=3/6), although the latter’s small sample size limits definitive interpretation.

Our results align with the growing body of evidence supporting NIV-NAVA as an effective NIV modality. Shin et al. [14] demonstrated in a prospective randomized controlled trial (RCT) that preterm infants (<30 weeks of gestation) randomized to NIV-NAVA following their first extubation had significantly lower failure rates within 72 hours compared to those on CPAP. Similarly, a retrospective study by Cheng et al. [15] in an Australian NICU reported an overall success rate of 86%. While our success rate of 58.3% is numerically lower, several factors could explain this discrepancy. First, our cohort was significantly smaller and characterized by a high prevalence of extremely low birth weight (ELBW) infants (82.1%) and a mean baseline RSS of 3.7, reflecting the respiratory disease burden at NIV-NAVA initiation. Second, given the retrospective nature of our study, a selection bias likely exists, as NIV-NAVA was frequently prioritized for infants with higher clinical complexity and more severe lung injury who had already failed or were expected to fail other NIV modes.

A compelling finding in our study was the high prevalence of active infection among episodes of NIV-NAVA failure. Among infants requiring subsequent intubation, 46.7% (n=7/15) had confirmed sepsis or pneumonia within the predefined peri-NIV-NAVA period. Previous studies support an association between infection and respiratory instability in preterm infants. Peng et al. [16] reviewed evidence suggesting that systemic inflammation and elevated inflammatory cytokines in preterm infants may influence respiratory control, while Kausch et al. [17] observed increases in apnea, periodic breathing, and bradycardia preceding late-onset sepsis in extremely preterm infants not receiving invasive mechanical ventilation. Such respiratory instability may increase respiratory support requirements and potentially contribute to NIV-NAVA failure despite the improved synchronization provided by NAVA. However, given the retrospective nature of our study, a causal relationship between infection and NIV-NAVA failure cannot be established.

Current literature, including the 2020 Cochrane meta-analysis, suggests that evidence remains insufficient to establish NIV-NAVA as superior for primary respiratory support at birth. However, more recent systematic reviews (Kuitunen et al.; Minamitani et al.) consistently indicate that NIV-NAVA significantly reduces reintubation rates when used as secondary support (post-extubation or escalation). Our findings support this trend, reinforcing the role of NIV-NAVA in preventing the cycle of extubation failure and its associated complications, such as barotrauma and volutrauma [8, 13-14, 18].

Despite these promising observations, the certainty of evidence remains low due to small sample sizes and heterogeneity across studies. The ongoing multicenter Phase III RCT by Matlock et al. is expected to clarify whether NIV-NAVA reduces extubation failure within a five-day window and to provide much-needed data on long-term outcomes, including BPD, neurodevelopmental injury, and mortality [19].

The main strengths of our study include the long-term evaluation (four years) in a real-world clinical setting and the detailed analysis of infection in relation to NIV-NAVA failure. However, limitations include the single-center retrospective design, the absence of a control group, and the small sample size. The lack of standardized protocols for NIV-NAVA initiation or intubation criteria reflects the pragmatic nature of our unit's practice but introduces inter-observer variability. Furthermore, the retrospective nature of the study limited the reliable assessment of apnea of prematurity, which was not a prespecified study variable. Future research should focus on larger prospective cohorts and specifically evaluate the potential role of NIV-NAVA in the management of apnea of prematurity, a promising area supported by emerging evidence.

## Conclusions

In summary, NIV-NAVA is a promising non-invasive ventilatory modality that enhances patient-ventilator interaction through optimal synchronization. Current evidence supports its safety and feasibility, demonstrating short-term clinical benefits such as reduced asynchrony, fewer desaturation episodes, and lower ventilatory pressures. Our study provides the first cohort description of NIV-NAVA application in a Portuguese NICU, reporting a clinical success rate in avoiding intubation that aligns with recent literature. These findings reinforce that NIV-NAVA may be particularly effective as a secondary respiratory support mode, especially in the post-extubation setting. While our results contribute to the growing body of evidence, the study's limitations underscore the necessity for larger, multicenter randomized controlled trials to definitively establish its efficacy and impact on long-term outcomes, including neurodevelopmental status, BPD incidence, and mortality rates in preterm infants.
